# Supplementary material for: Information-theoretic grid topology reconstruction using low-precision smart meter data
Source: Sci Rep. 2026 Jul 3;16:20547. doi: 10.1038/s41598-026-54490-4 (PMC13332023; doi:10.1038/s41598-026-54490-4)
Supplement: Supplementary file 1 — Supplementary Information. [file 41598_2026_54490_MOESM1_ESM.pdf]

# Supplementary Information: Information-Theoretic Grid Topology Reconstruction using Low-Precision Smart Meter Data

---

## S1 Additional Plots and Data

The additional plots shown here serve to better understand the model performance. The pairwise mutual information of the change in the current phasor for the SG1 dataset is seen in Figure S1. The behavior appears to follow an exponential decay model.

The SG1 dataset after the redundant nodes are removed is shown in Figure S2. Similarly, the SG2 dataset after the redundant nodes are removed is shown in Figure S3.

The histogram and the Gaussian approximation for the change in voltage magnitude from Node 33 in the SG1 non-solar dataset is shown in Figure S4. Note, the figure looks slightly different than other histograms in this work since we use Matlab's histcount function so that we can more easily compare values from the histogram approximation of the function to those from the Gaussian approximation.

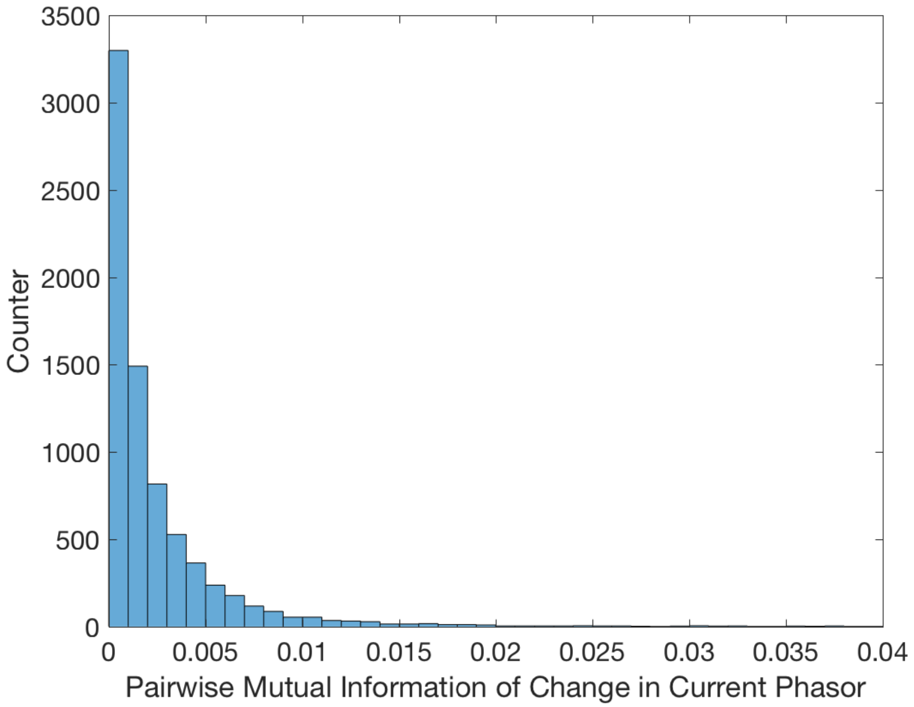


Figure S1: The pairwise mutual information of current phasor for the SG1 (non-solar) dataset.

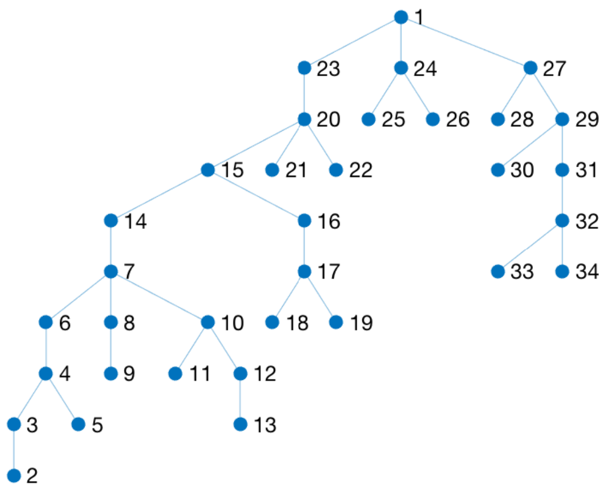


Figure S2: The true graph of the SG1 dataset once redundant nodes are removed. This is the graph the algorithm will estimate and with which its performance will be compared.

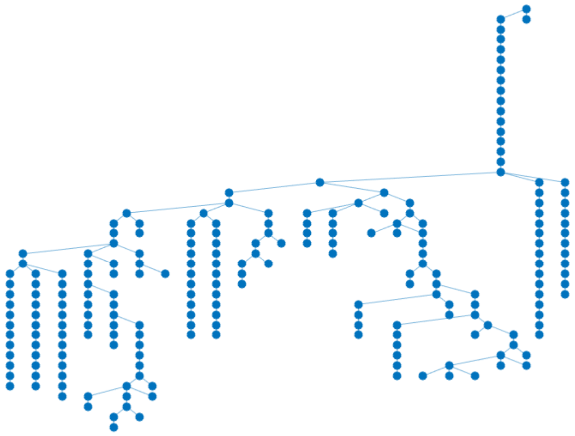

Figure S3: The true graph of the SG2 dataset once redundant nodes are removed. This is the graph the algorithm will estimate and with which its performance will be compared. Note, this graph has a special layout and the text labels are removed since the graph has so many nodes that it is difficult to fit in labels and use a non-compact layout.

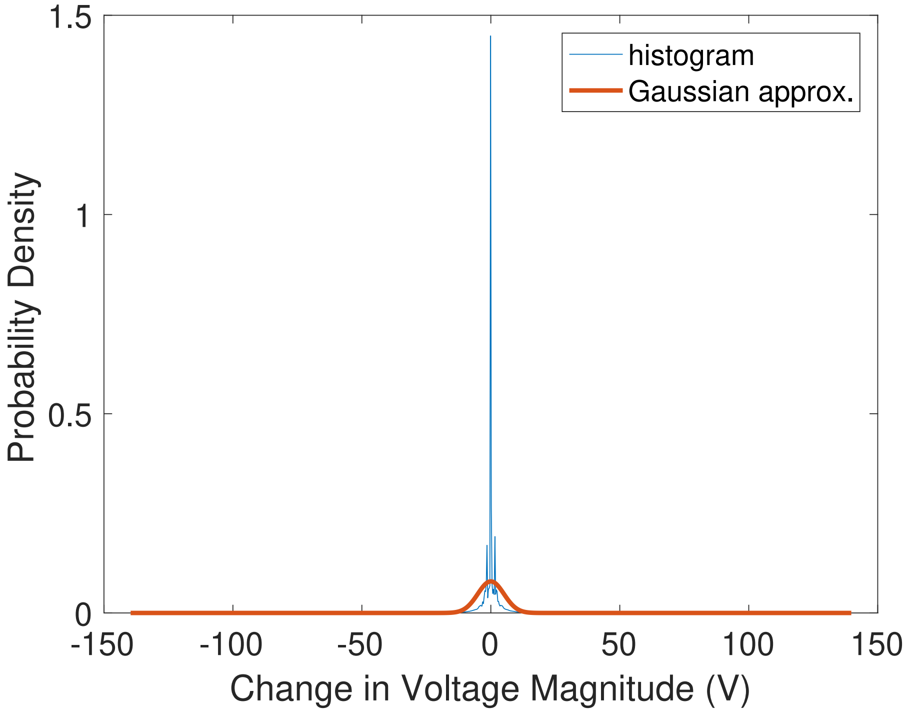


Figure S4: The histogram and Gaussian approximation for the change in voltage magnitude at Node 33 in the SG1 non-solar dataset. A thousand bins are used for the histogram.

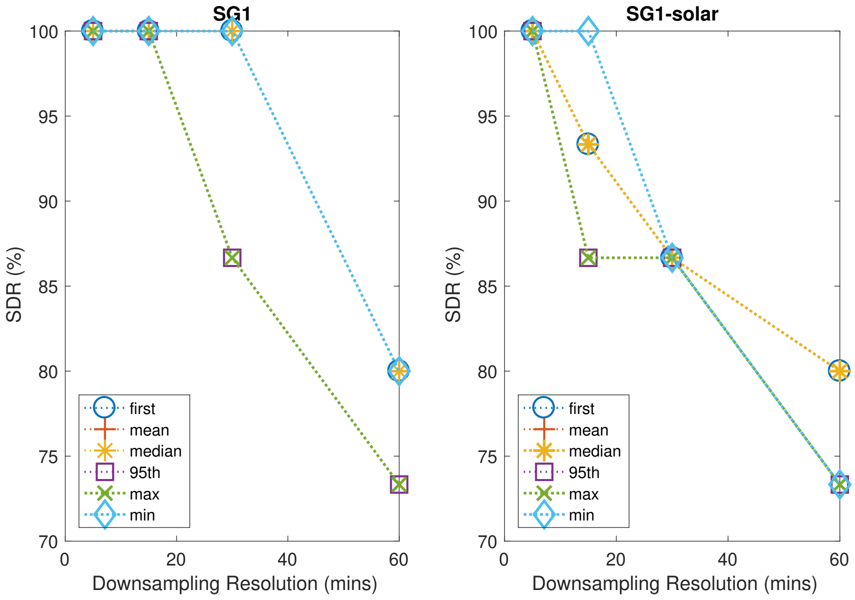


Figure S5: The SG1 dataset is down-sampled from the original dataset containing one sample per minute to 1 sample per 5, 15, 30 and 60 minutes and then the change in voltage magnitude of the dataset is taken before estimation. Different down-sampling methods are compared on the full year dataset.

Histograms and Gaussian approximations for node number 9 and 18 at different time resolutions in the SG1 (non-solar) dataset are shown in Figure S6.

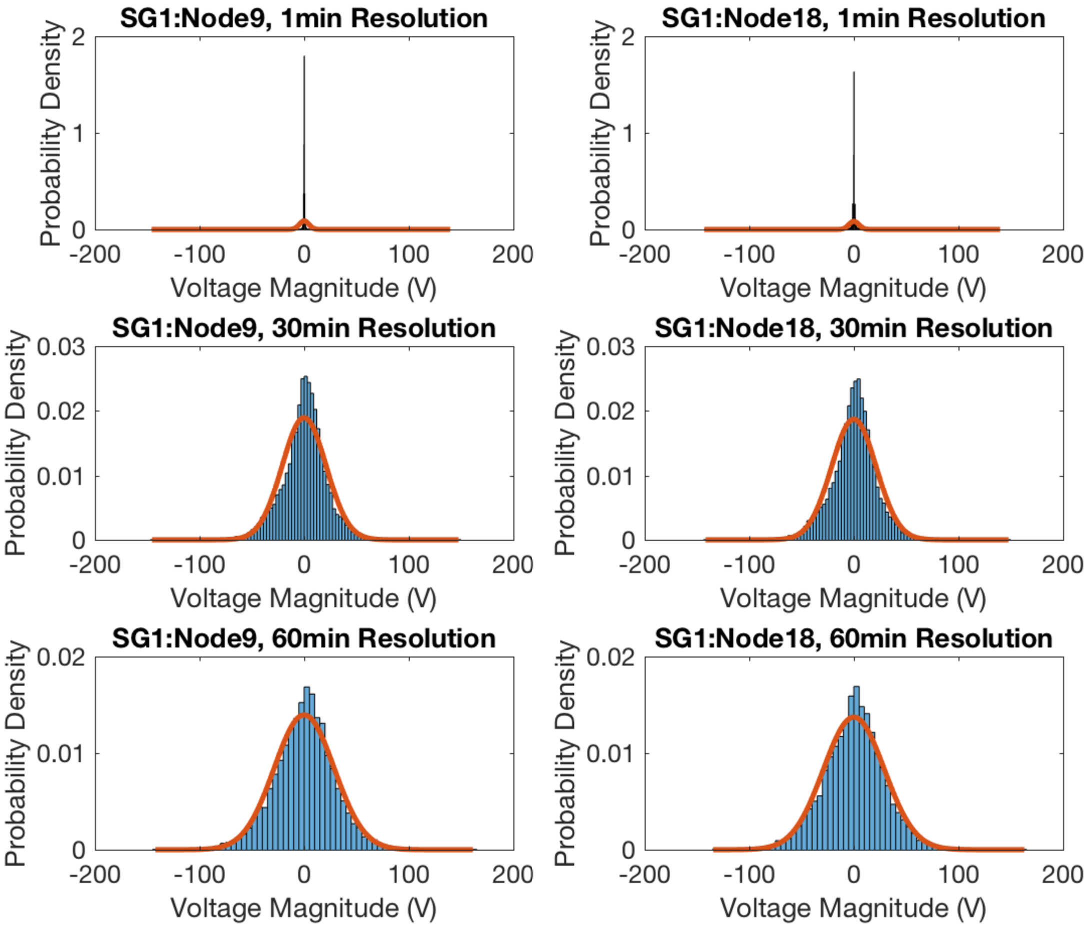


Figure S6: Histograms and Gaussian fits (line in red) for the SG1 (non-solar) dataset for nodes 9 and 18 at different resolutions. The derivative has taken for all resolutions.

We can better understand the Chow-Liu algorithm and how it ranks pairwise mutual information values to connect nodes with edges when reconstructing the power grid graph by looking at Figure S7. The figure shows the mutual information pairwise rankings as a function of downsampling resolution for Node 7 in the SG1 dataset when calculating the mutual information using a Gaussian approximation.

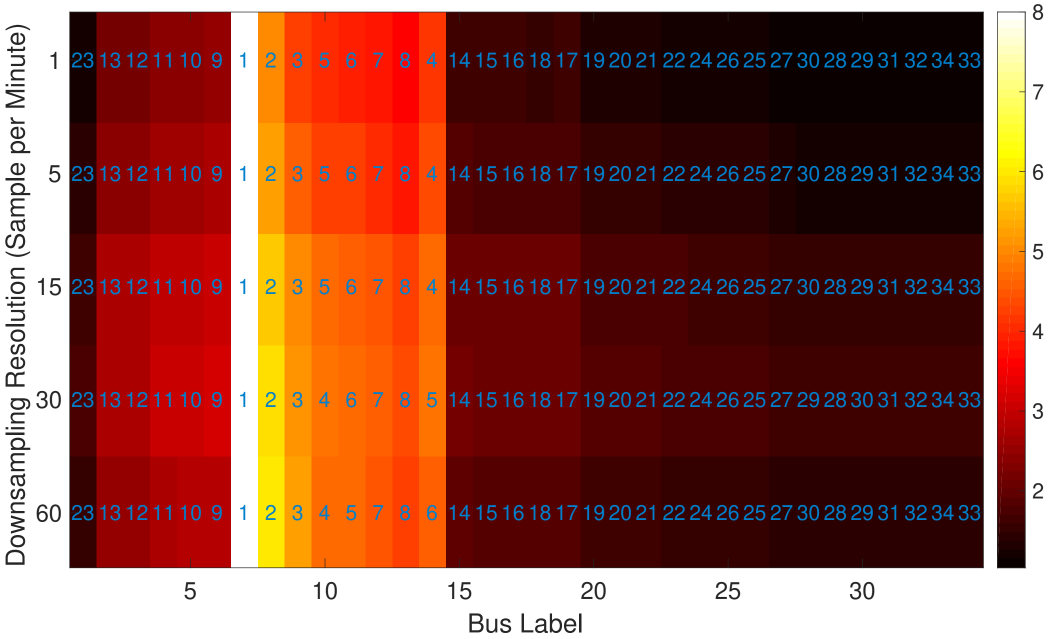


Figure S7: The mutual information pairing rankings as a function of downsampling resolution are shown as a heatmap for Node 7 in the SG1 dataset using the Gaussian mutual information on the change in voltage magnitude. The self-information, the mutual information between Node 7 and itself has the lowest ranking and conversely highest mutual information value for all resolutions. The resolutions shown are 1, 5, 15, 30 and 60 samples/minute.

Node 23's mutual information rankings in the SG1 dataset for the Gaussian approximations for different downsampling resolutions can be visualized in Figure S8.

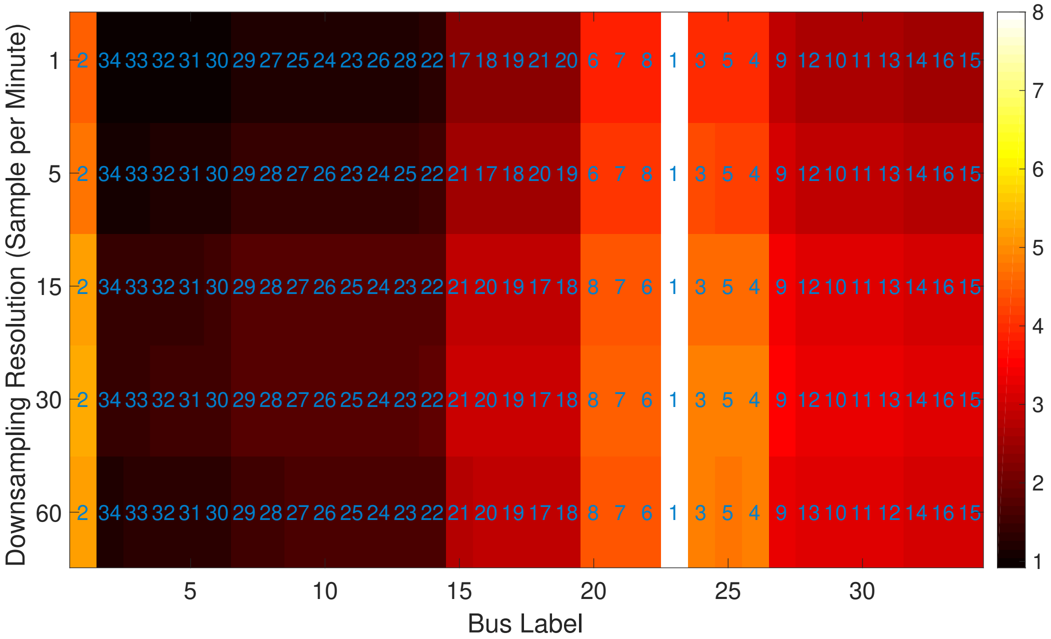


Figure S8: The mutual information paring rankings as a function of downsampling resolution are shown as a heatmap for Node 23 in the SG1 dataset using the Gaussian mutual information on the change in voltage magnitude. The self-information, the mutual information between Node 23 and itself has the lowest ranking and conversely highest mutual information value for all resolutions. The resolutions shown are 1, 5, 15, 30 and 60 samples/minute.

Figure S9 shows Node 23 for the SG1 solar dataset using the Gaussian approximation for different downsampling resolutions.

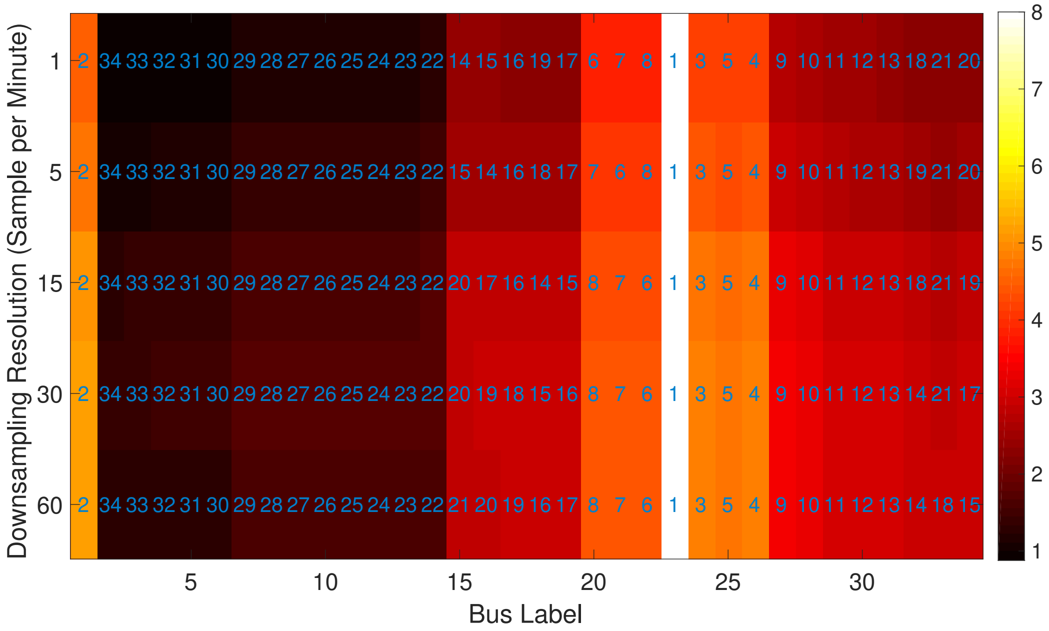

Figure S9: The mutual information paring rankings as a function of downsampling resolution are shown as a heatmap for Node 23 in the SG1 solar dataset using the Gaussian mutual information on the change in voltage magnitude. The self-information, the mutual information between Node 23 and itself has the lowest ranking and conversely highest mutual information value for all resolutions. The resolutions shown are 1, 5, 15, 30 and 60 samples/minute.

The SDR of the SG2 dataset when varying the downsampling rate for different methods of calculating the mutual information is seen in Figure S10.

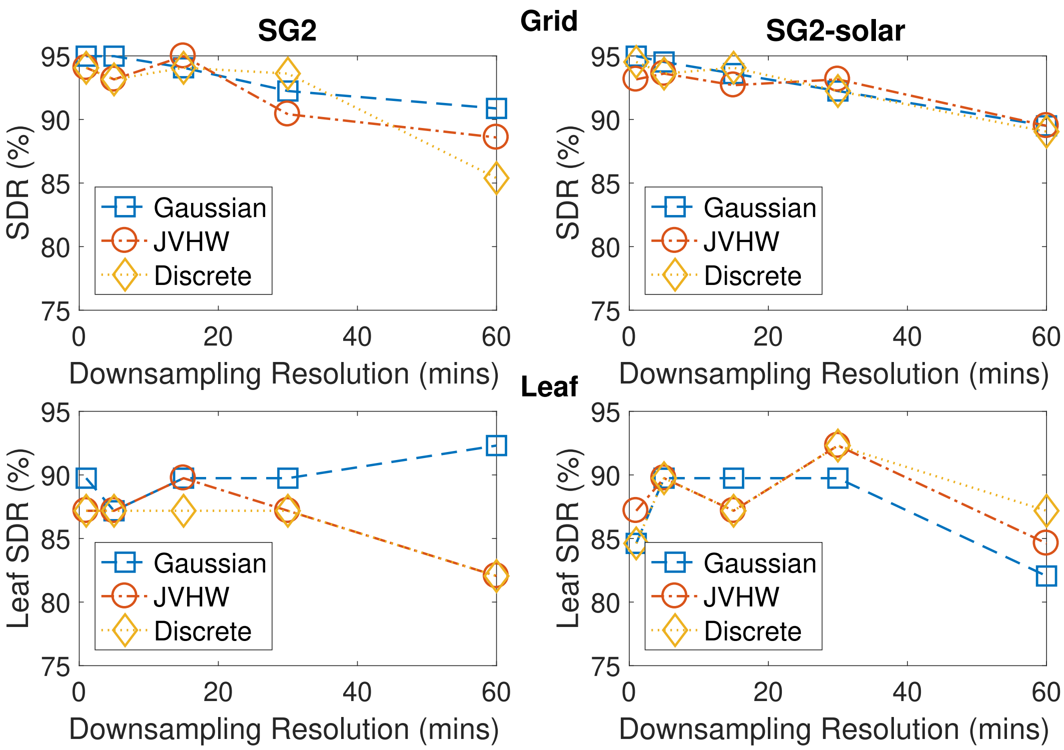


Figure S10: The SG2 dataset is down-sampled from the original dataset containing one sample per minute to 1 sample per 5, 15, 30 and 60 minutes and then the derivative of the dataset is taken before estimation.

The different time windows affect the SDR for the SG1 (non-solar) dataset are shown in Figure S11.

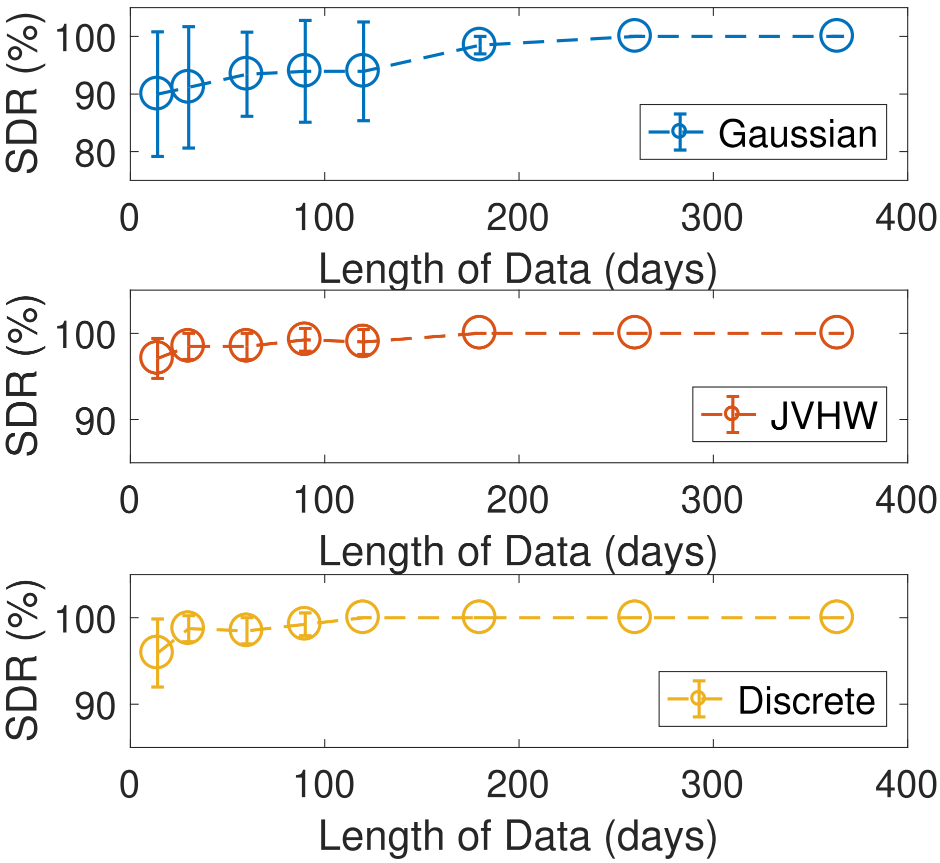


Figure S11: Lengths of consecutive days of data are taken of the SG1 (non-solar) dataset before estimation.

Figure S12 shows how the different time windows affect the leaf SDR for the SG1 (non-solar) dataset.

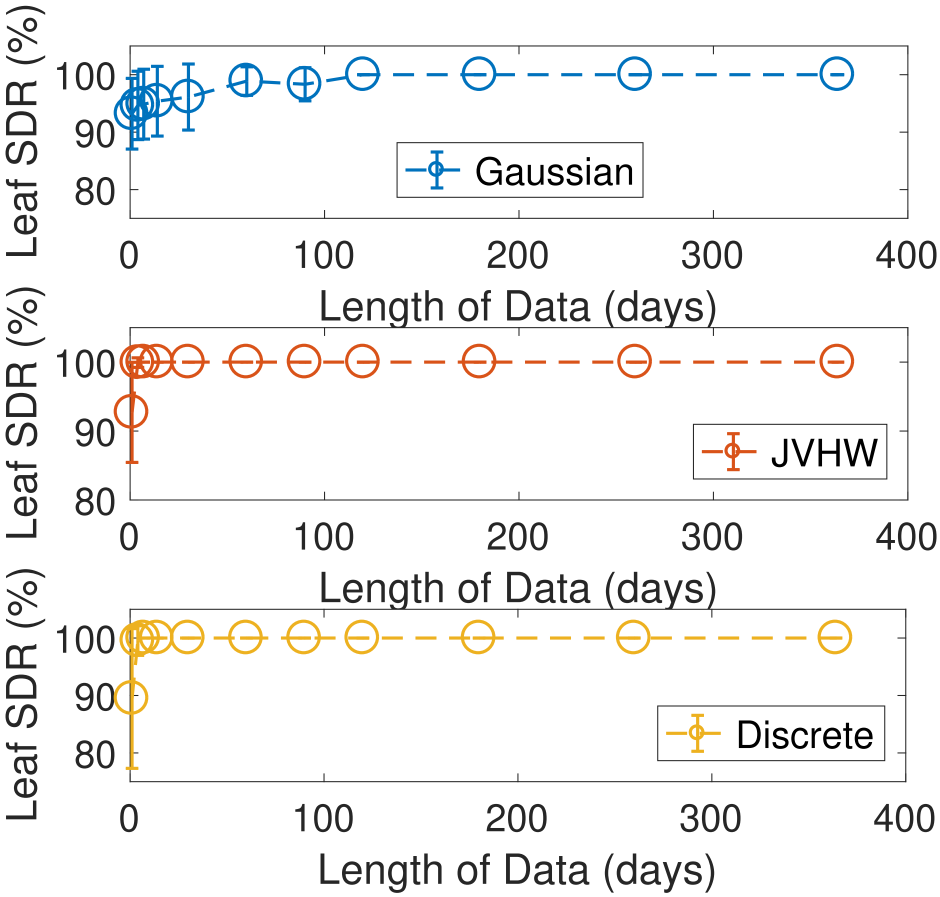


Figure S12: Different lengths of consecutive days of data are taken of the SG1 (non-solar) dataset before estimation.

The effect of a variable time window length on the leaf SDR for the SG1 solar dataset is seen in Figure S13.

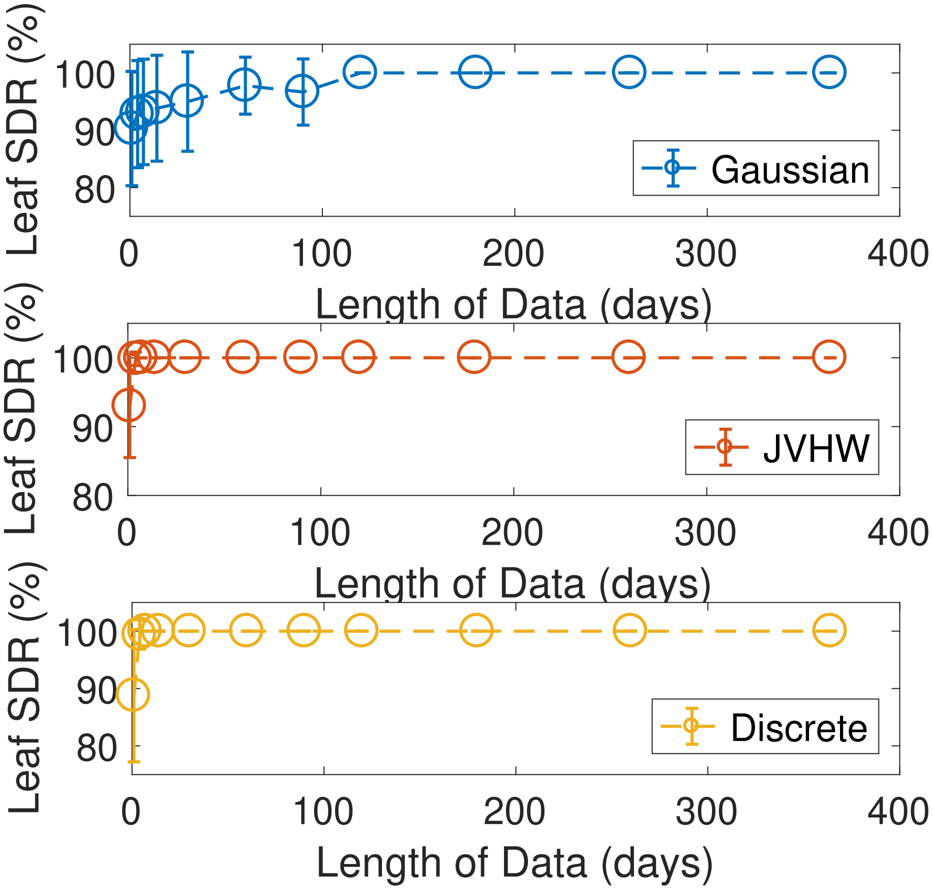


Figure S13: Different lengths of consecutive days of data are taken of the SG1 (solar) dataset before estimation.

Figure S14 shows how the different time windows effect the SDR for the SG2 (non-solar) dataset.

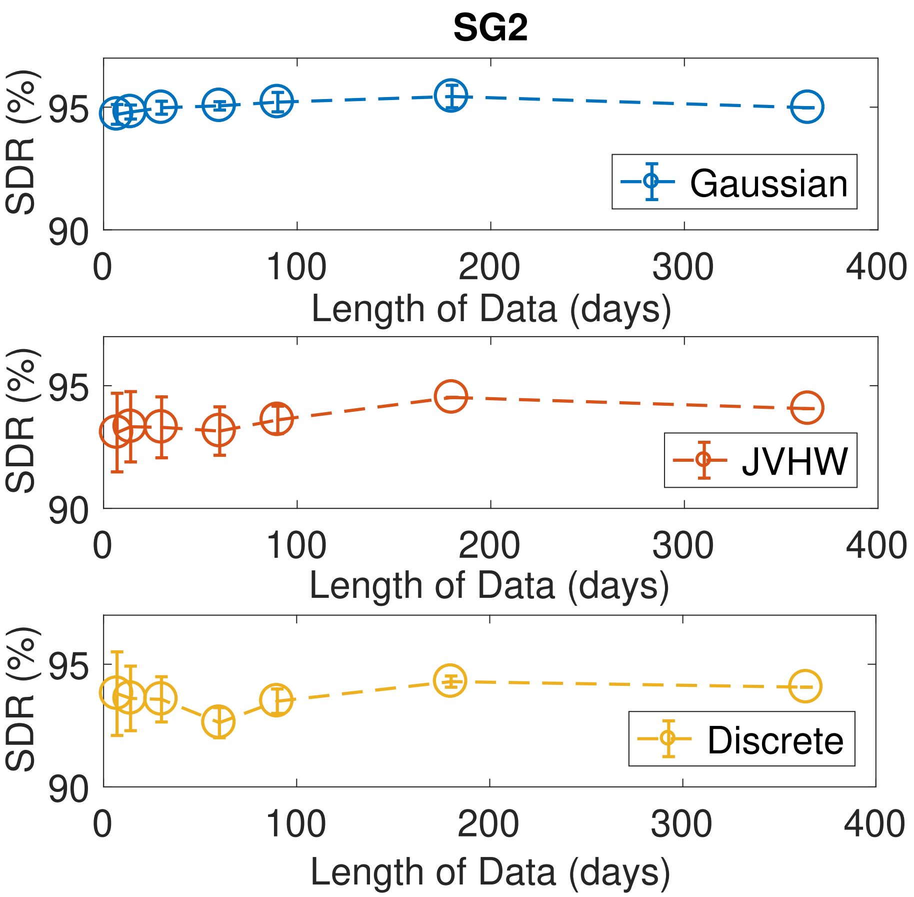


Figure S14: Different lengths of consecutive days of data are taken of the SG2 (non-solar) dataset before estimation.

Different time windows plotted against the SDR for the SG2 solar dataset are shown in Figure S15.

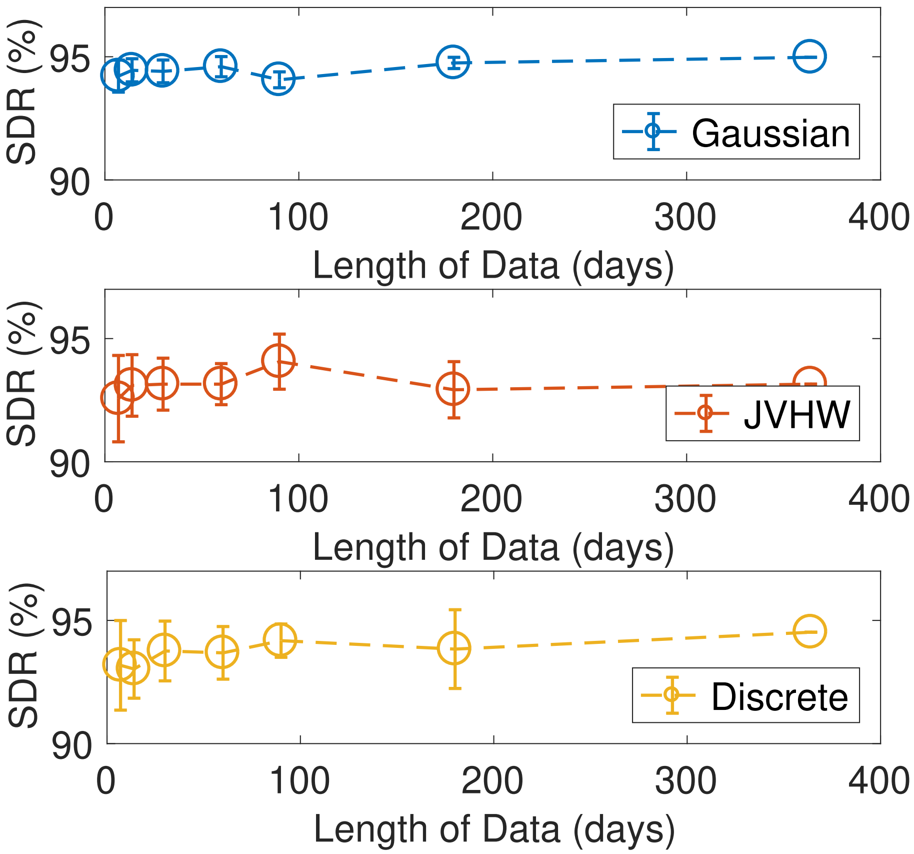


Figure S15: Different lengths of consecutive days of data are taken of the SG2 (solar) dataset before estimation.

Figure S16 shows how the different time windows effect the leaf SDR for the SG2 (non-solar) dataset.

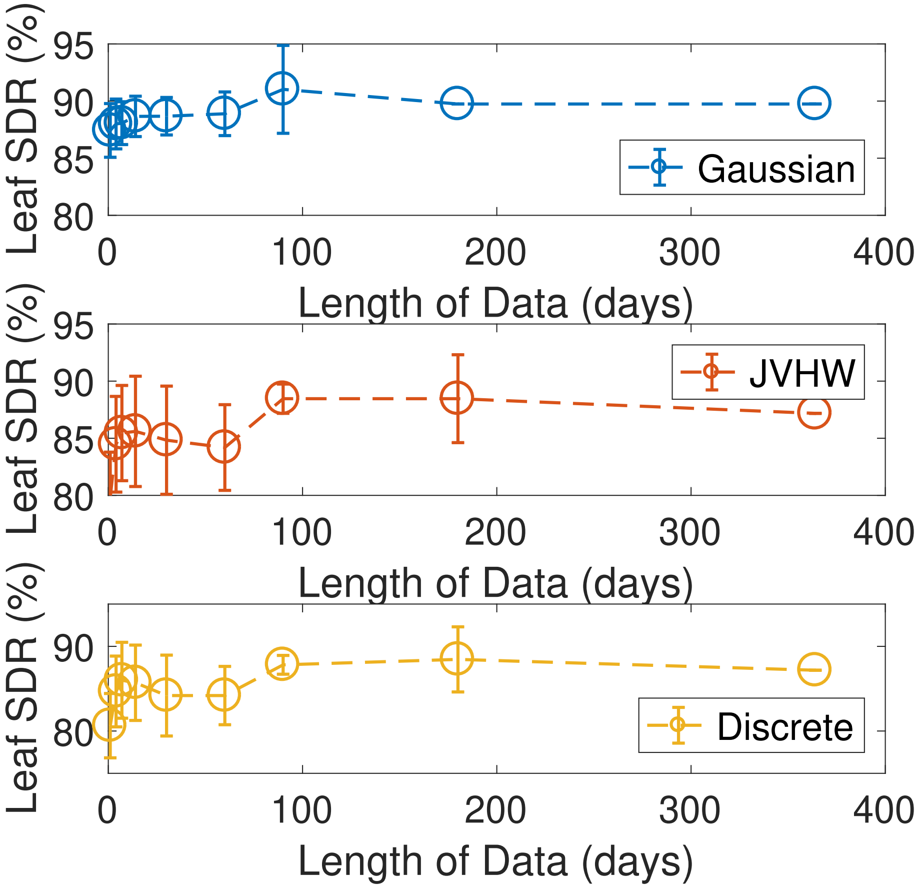


Figure S16: Different lengths of consecutive days of data are taken of the SG2 (non-solar) dataset before estimation.

The histogram and Gaussian approximation of the change in voltage magnitude for Node 15 of the SG2 solar dataset is shown in Figure S17. This node is consistently predicted to have an incorrect branch by the algorithm when using the Gaussian MI method for varying data lengths. This may be due to the Gaussian approximation being a poor fit as can be seen visually.

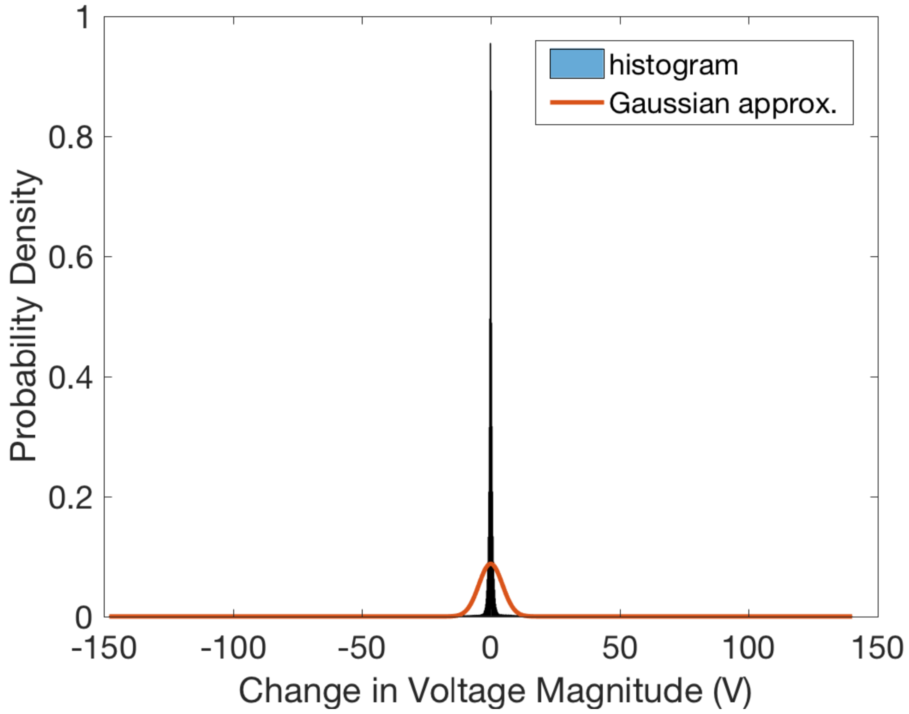


Figure S17: Histogram and Gaussian approximation of the change in voltage magnitude data at Node 15 of SG2 (solar) dataset.

Figure S18 shows voltage magnitude data discretized for the SG1 dataset and their associated Gaussian approximations for Node 9 (left) and Node 18 (right).

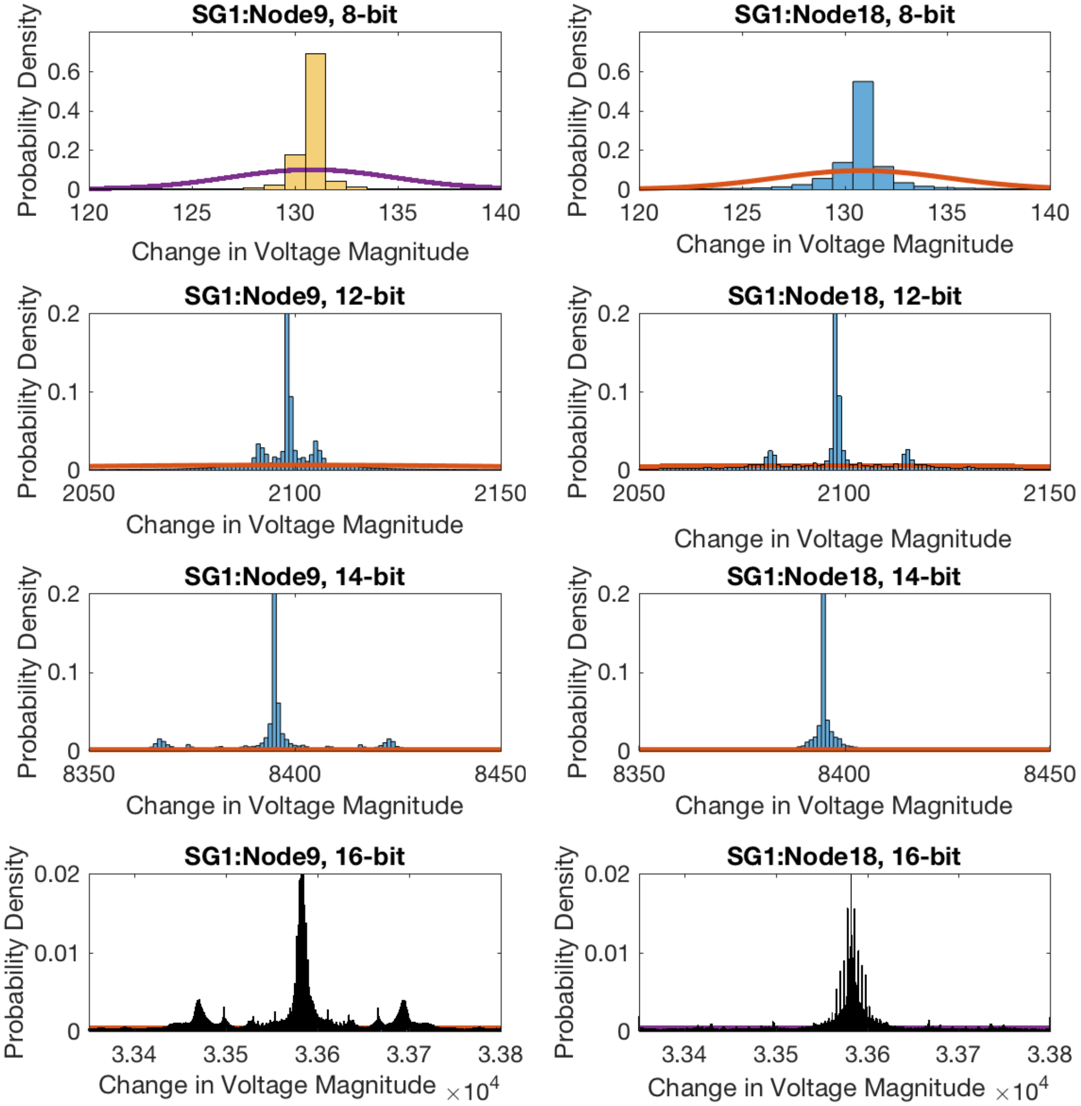


Figure S18: The incremental change in voltage magnitude for 1 minute step sizes is taken for the SG1 dataset. The data is then discretized to a variable size in bits. Gaussian approximations are shown overlayed.

The leaf SDR analysis is shown for 120 day time windows for the SG1 dataset in Figure S19.

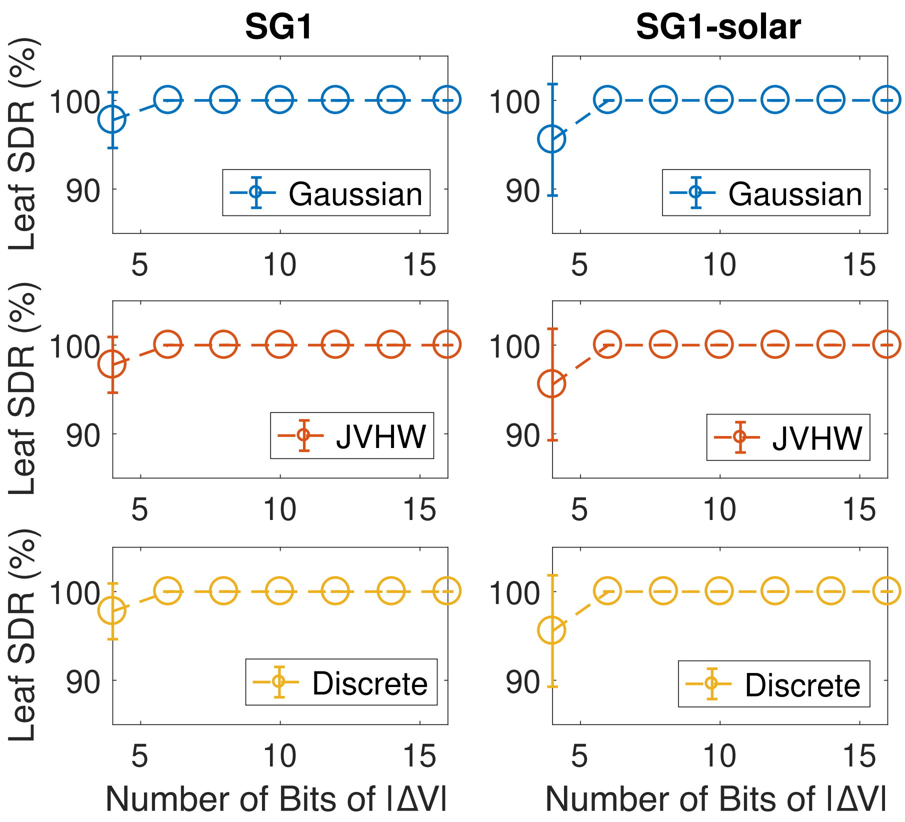


Figure S19: The SG1 dataset is discretized to a certain amount of bits and the leaf SDR is analyzed. Here a length of 120 days is used. The lengths of data are taken consecutively in the year-long dataset, meaning three sets of 120 days are used in the analysis.

The SDR analyzed is shown for the SG2 dataset in Figure S20. For a 120 day window it is shown in Figure S21.

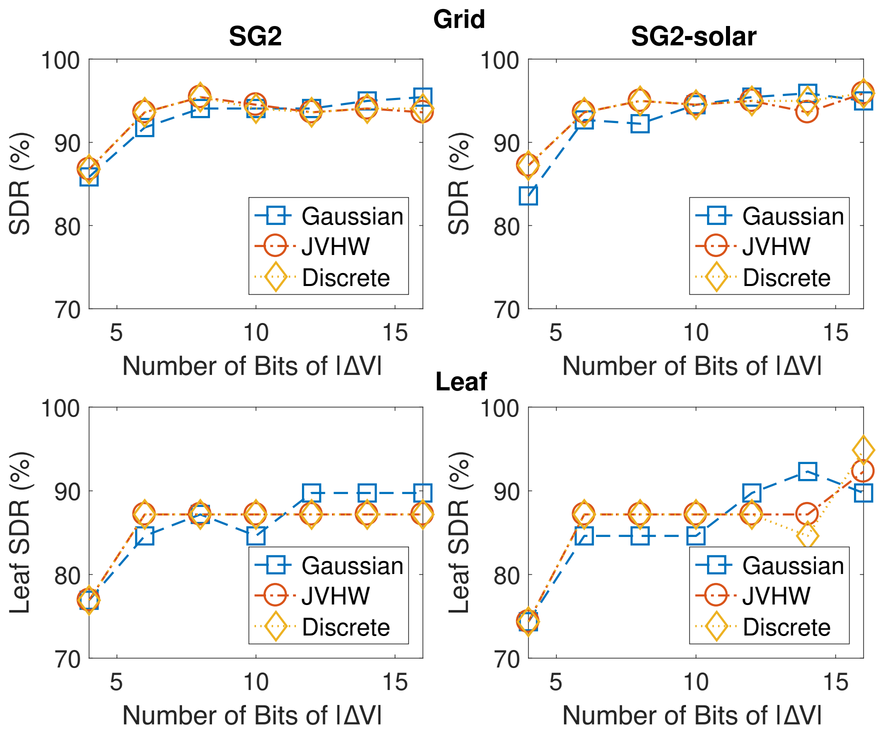


Figure S20: The SG2 dataset is discretized to a certain amount of bits. The entire dataset is used for each MI method.

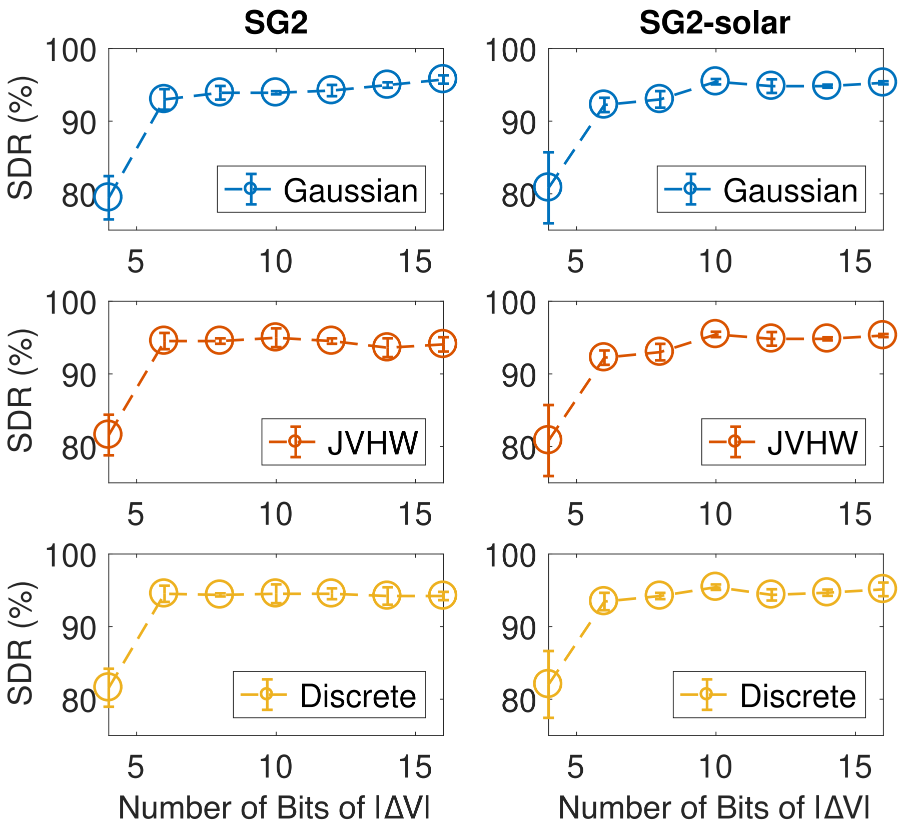


Figure S21: The SG2 dataset is discretized to a certain amount of bits. Here a length of 120 days is used. The lengths of data are taken consecutively in the year-long dataset, meaning three sets of 120 days are used in the analysis.

### S1.1 Varying the Time Step-size of the Change of Voltage Magnitude Variable

In this section we vary the time difference step size between voltage measurements and measure the effect on the algorithm's performance metrics. Equation (S1) shows a step size of 2 minutes (where time is given in units of minutes) when calculating the change in voltage magnitude.

$$|\Delta V_{\text{Step Size 2}}[t]| = |V[t]| - |V[t-2]| \tag{S1}$$

We can see in Figure S22 that both 34-node GridLAB-D datasets, SG1 and SG1 (solar), show significant drop offs in the SDR and leaf SDR for step sizes of 30 minutes or greater. The SDR drops off from around 100% to less than 90% for all MI methods for both SG1 datasets with 15 minute step-sizes. The discrete and JVHW methods estimate the SDR better than the Gaussian approximation as expected because the Gaussian approximation is a rather crude approximation for some nodes. The SDR plots show that change in voltage magnitude step sizes of greater than five minutes cause considerable degradation of estimation performance for the SG1 datasets.

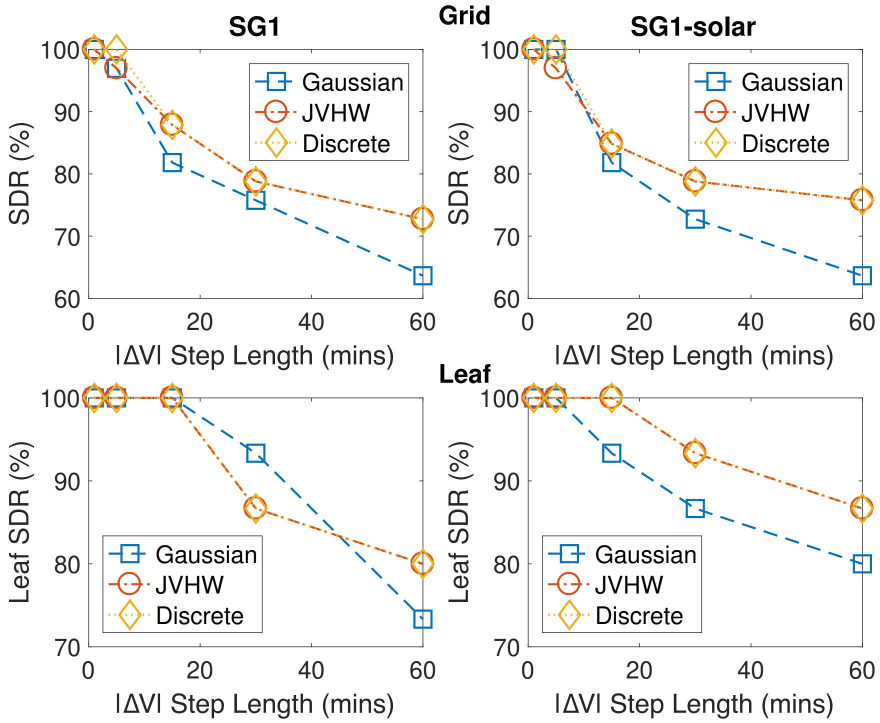


Figure S22: Variable change in voltage magnitude step sizes of the SG1 datasets plotted against the successful detection rate (SDR) and leaf successful detection rate. Note, magnitude is abbreviated to mag. in the axis label.

The leaf SDR plots show much better performance than the SDR plots in Figure S22 for all step sizes for the SG1 datasets. These datasets also show that fifteen minute step-sizes do not impact performance when using the discrete or JVHW MI methods. For the SG1 (non-solar) dataset, the Gaussian method outperforms the other MI methods by approximately 5% at a step size of 30-minutes. The SG1 solar dataset shows the Gaussian method outperforms other MI methods in terms of leaf SDR for all step-sizes greater than 5 minutes.

For the SG2 dataset, the Figure S23 shows the leaf SDR is lower than the regular SDR for variable changes in voltage magnitude step size. This is the opposite trend seen in the SG1 datasets. For the SG2 (non-solar) dataset the Gaussian MI method at a step size of 60 minutes performs much better than other methods in terms of leaf SDR. This is a trend seen in the resolution versus leaf node SDR experiments in the section below. In the SG2 (solar) dataset, the discrete and JVHW MI methods show improved SDR performance from a 1 minute step-length to a 5 minute step-length. The discrete and JVHW two MI methods also improve from a 30 minute step-length to a 60 minute step-length.

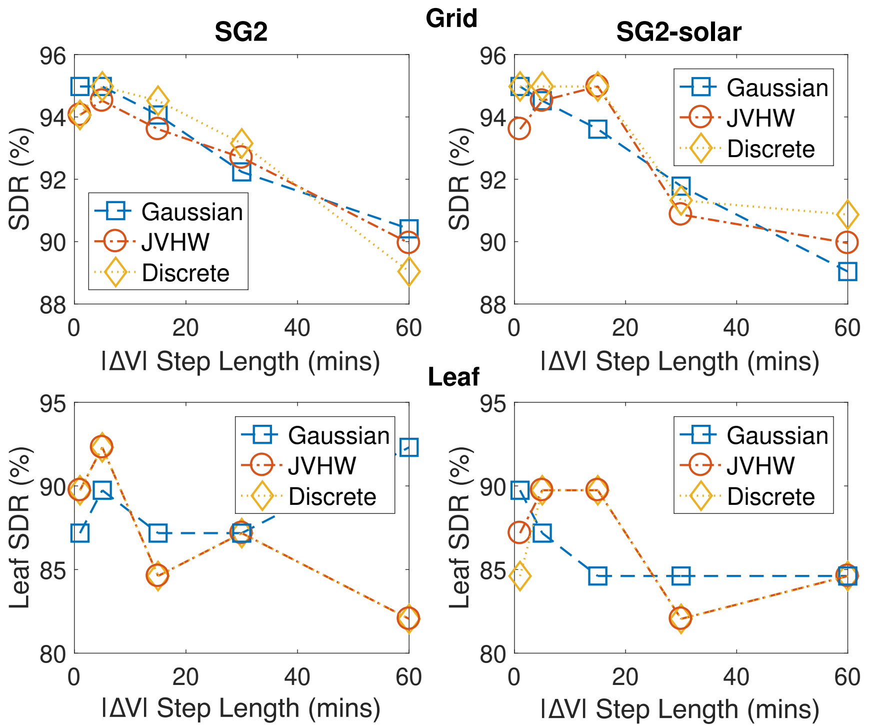


Figure S23: Variable step sizes for the change in voltage magnitude for the SG2 datasets plotted against the successful detection rate (SDR) and leaf successful detection rate. Note, magnitude is abbreviated to mag. in the axis label.

## S2 Kruskal's Algorithm

Kruskal's algorithm seeks to find the minimum weight spanning tree. A tree is a graph with no cycles. In a tree you cannot draw a path using connections between nodes on the graph from one node and back to the same node. Each connection is referred to as a pair of nodes $(x, y)$ in the graph and has an associated numerical value known as the weight $w(x, y)$ [1]. The minimum weight spanning tree is the graph which connects the nodes so that the sum of the weights of the connections is minimized so long as there are no cycles in the graph.

Kruskal's algorithm sorts all of the node pair weights in increasing order. The nodes with the minimum weight are connected. The algorithm then sequentially connects the next smallest weightings so long as the connection does not create a cycle in the graph. Implementations of Kruskal's algorithm keep track of the parent of each node. The parent or root of a node refers to the highest ranked node that is connected to the node in question.

There are several different implementations of Kruskal's algorithm, two of which differ in how they store the parent of a node [2]. When a pair of nodes is connected in the algorithm, one node is assigned as the parent of the other. This means one node has a higher ranking than the other. A node with four parents could be referred to as a rank four node. Alternatively, if a node has more than one parent, the path to the lowest ranked node can be compressed where the algorithm only keeps track of the lowest ranked parent for each node.

When adding a connection in the graph in Kruskal's algorithm we need to be sure the connection will not cause a cycle. If two nodes have the same parent node then a cycle will be formed if these nodes are connected. Thus, the algorithm, before adding a connection, checks the lowest-

ranked parent of each node about to be paired. The path compression implementation improves performance in this step of the algorithm.

Matlab has its own min-span-tree function but it requires the bio-informatics toolbox which many labs do not have. In order to increase usage of the grid topology estimation algorithm we wrote our own script to avoid licensing issues. Our implementation performs 10x slower than Matlab's version as seen in Table S1. Since Kruskal's algorithm execution time is negligible compared to mutual information computation time (as shown in Table S1), minimal effort has been placed to improve our implementation of Kruskal's algorithm.

| **Implementation** | **Mean Performance (s)** |
|---|---|
| Our Implementation | 0.0154 |
| Matlab | 0.0017 |

Table S1: Timing performance for Matlab's and our implementation of Kruskal's algorithm on the random power factor IEEE-123 Node network. The mean of three runs was taken.

## References

[1] Thomas H. Cormen, Charles E. Leiserson, Ronald L. Rivest, and Clifford Stein. *Introduction to Algorithms*. MIT press, 4th edition, 2022.

[2] Aaron Kershenbaum and Richard Van Slyke. "Computing minimum spanning trees efficiently." *Proceedings of the ACM annual conference*, Volume 1, pp. 518–527, 1972.
